# Current status and challenges in lumbar proprioception measurement: a narrative review

**DOI:** 10.3389/fneur.2026.1747474

**Published:** 2026-06-29

**Authors:** Xiansheng Zhao, Maoqing Fu, Lihe Li, Chaoliang Lv

**Affiliations:** 1 Department of Graduate, Shandong First Medical University, Shandong Academy of Medical Sciences, Jinan, China; 2 Department of Spinal Surgery, Jining NO.1 People's Hospital Affiliated to Shandong First Medical University, Shandong Academy of Medical Sciences, Jining, China

**Keywords:** lumbar, measurement, motion sense, position sense, postural balance, proprioception

## Abstract

Lumbar proprioception is the cornerstone for maintaining dynamic spinal stability, postural control, and central sensorimotor regulation. Clinically, proprioceptive deficits are closely tied to the occurrence and recurrence of degenerative lumbar diseases and chronic non-specific low back pain (CLBP). Despite its clinical relevance, objectively and accurately quantifying this metric remains highly controversial methodologically. This review provides a structured overview comparing existing evaluation tools, mapping their technical characteristics and clinical feasibility. To ensure a comprehensive evaluation, a literature search was conducted via PubMed and Web of Science databases utilizing Medical Subject Headings (MeSH) terms related to “lumbar spine” and “proprioception,” supplemented by keywords including “motion sense,” “vibration sense,” and “force sense.” Among direct metrics, position sense via inclinometer-based active joint repositioning is most widely applied, whereas motion, force, and vibration senses are less utilized. Within position sense tracking, common clinical tools like dual inclinometry and tape measurement demonstrate weak concurrent validity, large errors, and low test–retest reliability, with absolute reliability further obscured by the lack of a “gold standard.” Furthermore, distinct direct metrics (motion threshold, active repositioning, and passive repositioning) lack significant inter-modal correlation. While postural balance offers a more holistic analysis of lumbar function than isolated components, its application remains limited as it is highly susceptible to confounding factors and fails to isolate or directly represent localized peripheral mechanoreceptor proprioception. In conclusion, although a multitude of methodologies have emerged, the inclinometer-based active joint repositioning paradigm demonstrates the highest feasibility in routine clinical practice. However, a universally accepted gold standard remains persistently absent, and objective quantification requires further comprehensive exploration.

## Introduction

Proprioception, also known as the “sixth sense” or muscle sense, was first proposed by Charles Sherrington in relation to motor control (1). Sensory receptors are primarily located in muscles, tendons, and joints, including muscle spindles within skeletal muscles, Golgi tendon organs at the muscle-tendon junction, and low-threshold mechanoreceptors (e.g., Ruffini endings, Pacinian corpuscles) within joint capsules. By perceiving information about joint and body positions, spatial movement, and muscle forces, proprioception provides accurate data about both the external and internal environment of the body, supporting effective motor control (2, 3). While it serves as a critical afferent input for motor regulation, this does not imply that it can independently coordinate motor outputs. Instead, sensorimotor control represents a higher-order, overarching system that integrates these localized proprioceptive signals with visual, vestibular, and tactile feedback to execute coordinated motor control (4).

Currently, an increasing number of spinal surgeons and researchers are focusing on the importance of lumbar proprioception. Specifically, lumbar proprioception denotes the regional position and movement awareness required for spinal segmental stability (5). In clinical settings, current literature demonstrates a clear association between lumbar proprioceptive deficits and the development or persistence of chronic nonspecific low back pain (6–8). As a plausible mechanism, it is hypothesized that acute lumbar pain prompts altered movement patterns, and this impaired motor control subsequently alters spinal loading, potentially leading to spinal and neural imbalances that exacerbate load and promote neuroplastic changes contributing to pain chronicity (9, 10). Proprioceptive dysfunction is also observed during rehabilitation following lumbar spine surgery, where postoperative local tissue damage—particularly to muscles, ligaments, and joint capsules—is linked to altered postural perception and decreased stability of the lumbar spine (11–13). While these issues are thought to interfere with the patient’s ability to accurately control spinal movements and execute effective motor patterns during recovery (14, 15), or reinforce incorrect movement habits that increase re-injury risks (16, 17), the direct demonstrated clinical effects of isolated proprioceptive impairment remain a critical area of ongoing evaluation. Therefore, assessing and restoring lumbar proprioception is crucial in clinical management and rehabilitation.

To address this gap, the primary objective of this review is to compare the psychometric properties (reliability and validity) of available tools, map their technical characteristics, and determine their clinical feasibility. Specifically, this review explicitly aims to answer the following research question: What are the comparative psychometric properties and clinical utilities of current lumbar proprioception measurement methods across healthy and clinical populations? To resolve this fundamental inquiry, this article provides a structured and multidimensional review of lumbar proprioception assessment. Active and passive methodologies for lumbar position sense are primarily analyzed to evaluate their respective reliability and clinical utility. Current measurement techniques for motion, vibration, and force sense are then systematically reviewed. Indirect inference approaches for lumbar proprioception utilizing postural balance analysis are also explored. Ultimately, the discussion section analyzes the inherent challenges and key limitations confronting these aforementioned methodologies.

## Methods

A literature search was primarily conducted via the PubMed and Web of Science database, utilizing Medical Subject Headings (MeSH) terms related to “lumbar spine” and “proprioception.” Supplementary keyword searches were further performed using terms including “Motion Sense,” “Vibration Sense,” and “Force Sense” to ensure a comprehensive evaluation. Regarding the assessment of lumbar position, motion, vibration, and force sense, the inclusion criteria were strategically focused on novel, contemporary, and widely adopted methodologies, while obsolete or outdated techniques lacking current clinical relevance were excluded. Similarly, for postural balance analysis, the selection was strictly confined to protocols commonly utilized within the clinical practice of spinal surgery, whereas all other non-relevant methods were excluded.

## Results

### Lumbar position sense

Position sense measurement is considered a direct indicator of lumbar proprioception (18). Position sense is typically evaluated using active or passive repositioning tests, both requiring subjects to reproduce a previously experienced position. Participants typically perform slow, continuous movements at a uniform speed to reach the position, and the degree of deviation from the target position reflects the extent of impairment in lumbar joint position sense across both healthy populations and clinical cohorts.

To comprehensively evaluate lumbar joint position sense, it is essential to distinguish among various error measurement techniques, each capturing a different dimension of proprioceptive performance. (1) Absolute Error (AE) is the primary metric indicating overall repositioning accuracy without regard to direction. (2) Constant Error (CE) accounts for the direction of the deviation, revealing any systematic bias (a tendency to consistently overshoot or undershoot the target angle) (19). (3) Variable Error (VE) measures the subject’s consistency or directional variability across multiple trials, serving as an indicator of motor control stability. (4) Root Mean Square Error (RMSE) quantifies overall system performance; this metric is highly sensitive to and heavily penalizes larger measurement deviations, particularly those near the extremes of the range of motion. (5) Absolute Constant Error (|CE|) calculates the absolute value of the average CE, providing a measure of overall response bias magnitude while avoiding the confounding effect of opposing directional errors canceling each other out during averaging. (6) Total Error (E) incorporates both systematic bias and response variability, serving to evaluate the total spread of movements around the actual target. Although these metrics capture distinct aspects of proprioception, AE is universally accepted as the most direct measure of repositioning accuracy, where lower values consistently reflect superior lumbar joint position sense (20).

#### Active joint repositioning test

The active joint repositioning test involves the subject moving autonomously from an initial position to a remembered target angle or position without visual or auditory cues (21). After familiarization with the target, the subject returns to the initial position and then actively moves the lumbar spine to the perceived target location, where positional deviations are recorded to calculate error metrics. From a psychometric perspective, while the fundamental underlying principle for evaluating joint position sense remains consistent; the variations lie primarily in the measurement tools employed, such as inclinometers, tape measures, or other devices.

From a biomechanical perspective, it is well documented that repositioning errors are inherently higher near the extreme limits of the range of motion (ROM) due to maximal capsuloligamentous tension and altered proprioceptive feedback (22, 23). Consequently, standard protocols for these tools universally adopt specific target positions to avoid repositioning errors caused by extreme positions. For instance, the dual-inclinometer test typically sets the target angle precisely at 50% of the subject’s full lumbar ROM to evaluate repositioning accuracy within a range of heightened proprioceptive sensitivity. By comparison, tape measurement provides a surface-distance alternative (24, 25). Instead of relying on a ROM percentage, it utilizes a fixed displacement, such as a 5 cm increase between the C7 and S1 spinous processes during active flexion. Ultimately, whether employing relative angular percentages or absolute surface distance increments, both protocols adhere to the identical fundamental procedures described above, yet this methodological variance directly impacts the comparability of baseline data across different clinical studies.

**Figure 1:**
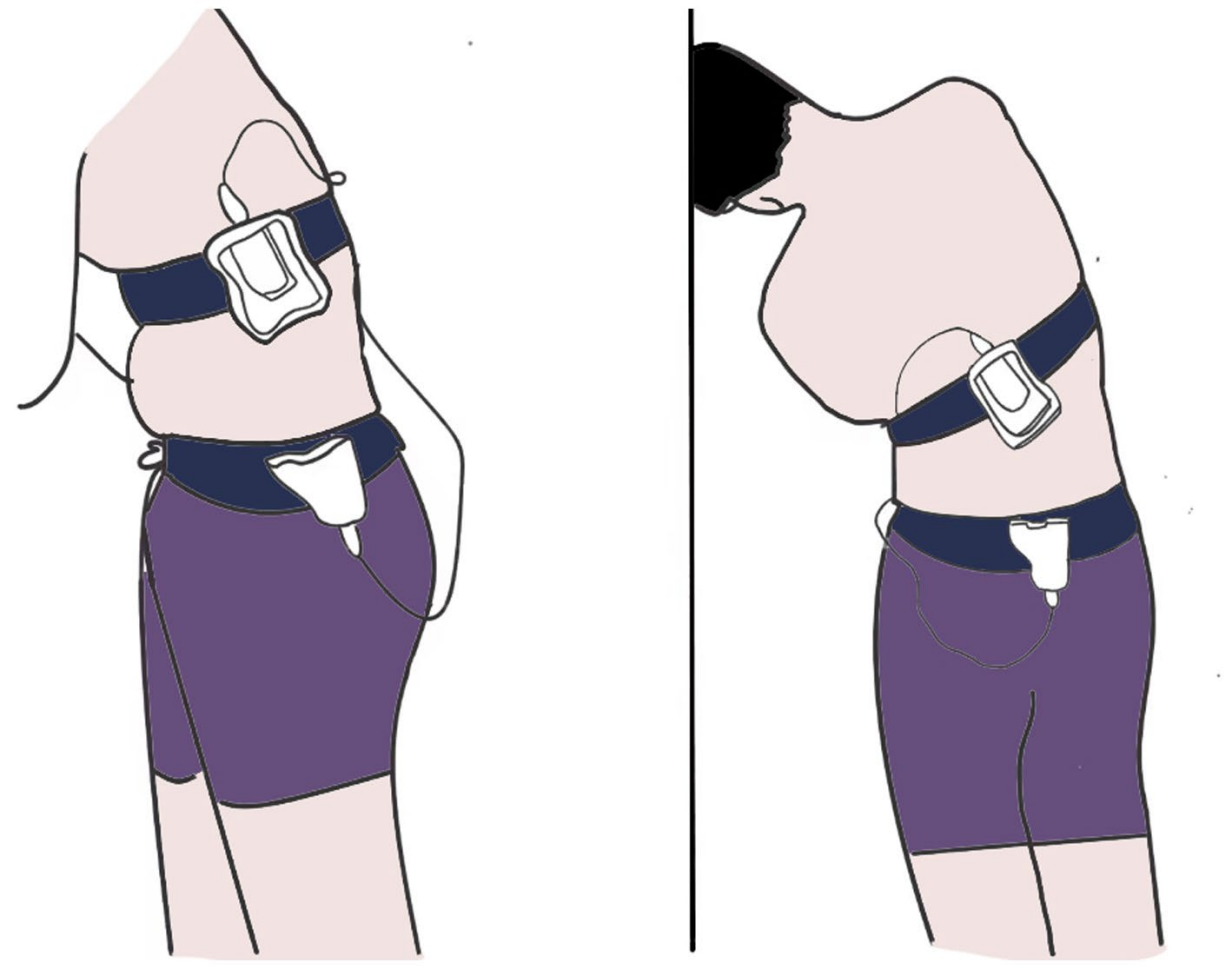
During the assessment, subjects were blindfolded to ensure visual occlusion, while the environment was kept quiet to minimize auditory interference. Participants were instructed to maintain a neutral standing posture with their knees fully extended and weight evenly distributed on both feet. For the evaluation of lumbar flexion repositioning errors in the sagittal plane, the primary sensor (digital inclinometer) was placed laterally at the T12 level, and the secondary sensor was positioned at the S1 level of the hemipelvis. Conversely, to record lateral bending errors in the frontal plane, the primary and secondary sensors were placed at the T12 level and the sacral midpoint, respectively (32).

The inclinometer is the most used device in active repositioning measurement methods (26–28). Given the limited accessibility and poor anatomical adaptability of traditional digital inclinometers, smartphone applications integrating inclinometer functions have recently been developed. These applications offer a rapid, accessible, and convenient alternative for measuring proprioceptive errors and have become widely adopted in routine lumbar assessments (29, 30). During the testing procedure, a dual-inclinometer system is typically employed to isolate lumbar movement from pelvic tilt. The primary inclinometer is placed at the T12 level of the thoracic spine, and the secondary inclinometer is positioned at the S1 level of the sacrum. During the assessment, the target position is usually set at 50% of the subject’s full lumbar range of motion (ROM), or at a specific target lumbar position for precise testing (Figure 1). When appraising the psychometric properties of this approach, a stark inconsistency in test–retest reliability emerges between different target populations. Traditional dual-inclinometers generally demonstrate moderate to excellent intra-rater reliability (ICC = 0.73–0.91, SEM = 0.36–1.31°) in evaluating lumbar joint position sense (31), with good agreement observed in specific postural tests such as neutral or target lumbar positioning (ICC = 0.75–0.93) (32). However, their measurement reproducibility exhibits significant clinical divergence: while reliability remains stable in asymptomatic populations (ICC = 0.76–0.80), it drastically degrades in patient groups, such as chronic low back pain or post-surgical cohorts (ICC = 0.31–0.64) (33). This evidence underscores a critical methodological limitation: traditional inclinometry is highly operator-dependent and susceptible to the altered neuromuscular control characteristic of clinical pathologies. In contrast, smartphone-based measurement applications demonstrated outstanding stability, exhibiting moderate to excellent test–retest reliability (ICC = 0.67–0.96) (34, 35).

The tape measure is less prevalent than inclinometers in proprioception studies, possessing a similar underlying principle in assessing lumbar joint position sense. The primary measurement tool is a standard or plastic tape measure, used to quantify the surface distance between specific spinal anatomical landmarks. Currently, it is widely recognized as a simple, flexible, inexpensive, and highly accessible clinical assessment method (36). Despite its high accessibility, a profound psychometric gap in the current literature is the lack of rigorous validation data confirming its strict test–retest reliability and construct validity across pathological cohorts. Furthermore, a critical methodological limitation of both these techniques is their inability to capture three-dimensional spinal kinematics. Relying on these surface-based tools, evaluators can only report localized angular changes or linear distances between skin markers, and cannot account for the inherent complex, multi-planar motions and coupled rotations during lumbar repositioning, which potentially compromises the validity of measurements in patients with structural spinal deformities.

Isokinetic dynamometers are considered highly effective and reliable tools for proprioception assessment (37). Standardized testing protocols typically require subjects to be seated with the mechanical arm axis aligned with the L5-S1 intervertebral disc space, executing active repositioning to predetermined target angles (e.g., 30° and 60° of flexion) across repeated trials to compute mean errors (38–40). Although data extrapolated from peripheral joints suggest excellent intra-rater reliability (ICC = 0.91–0.96) (41), direct psychometric validation within dedicated lumbar clinical cohorts remains scarce. A primary advantage of the isokinetic dynamometer is its capacity to provide highly controlled, standardized, and objective kinematic data. However, several inherent limitations must be acknowledged in relation to its clinical utility. Crucially, the system typically measures repositioning accuracy exclusively in the sagittal plane, which restricts its utility in assessing lumbar proprioception during lateral bending and rotational movements. Furthermore, the system’s resolution cannot quantify the fractional portion of the angle, which may affect measurement precision. Lastly, the physical attachments required to secure the subject may introduce extraneous skin sensory input, creating a confounding tactile feedback loop that theoretically threatens the construct validity of pure proprioceptive testing (42). Although some evidence suggests that altered skin sensitivity has a minimal impact on motor performance during active tasks in healthy individuals (43). its confounding effect in desensitized post-surgical or neuropathic patient populations remains completely unanswered.

**Figure 2:**
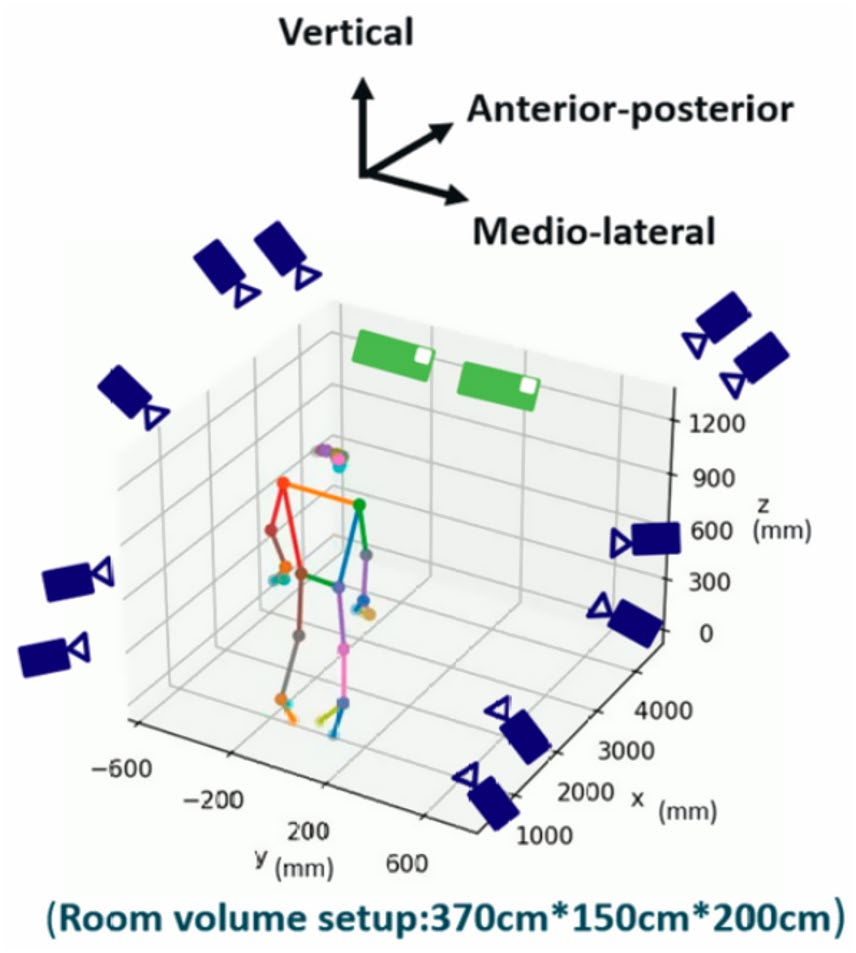
Motion data acquisition was conducted using an 11-camera VICON system in conjunction with two iPhone 14 smartphones. Specifically, the VICON system (sampling rate: 120 Hz) utilized 45 reflective markers to capture precise joint kinematics. In parallel, the MediaPipe system estimated 3D poses from 2D images captured by the dual iPhone 14 cameras, providing a markerless alternative for spatial posture analysis (47). This figure is reproduced directly under the open-access CC BY license.

Electronic motion capture systems represent a highly reliable methodology for the objective assessment of joint position sense (44). While these systems initially utilized electromagnetic sensors to evaluate lumbar proprioception (45), current research and clinical practices have largely transitioned toward the use of sophisticated three-dimensional (3D) kinematic motion capture systems. Contemporary protocols utilize retroreflective markers combined with spinal curvature algorithms to track precise trajectories and compute multi-planar AE (46). Empirically, these high-resolution systems demonstrate strong discriminative validity, successfully differentiating the complex kinematic deviations of patients with degenerative lumbar diseases from those of healthy controls (Figure 2) (47). The fundamental principle of this approach is essentially similar to the use of electronic motion capture systems for assessing various lumbar positions. Although reliable and effective, these systems lack portability, are time-consuming to set up, and require specialized software for analysis (48), Consequently, a significant remaining gap in the field is the lack of clinical feasibility for routine screening.

#### Passive joint repositioning test

The passive joint repositioning test assesses lumbar proprioception by utilizing a motorized device to move the subject’s spine to a predetermined target position. Similar to active repositioning, the subject is first familiarized with the target angle. However, the fundamental difference lies in the execution: rather than the subject actively initiating the movement, the device drives the trunk at a constant, highly controlled speed. Once the subject perceives that the target position has been reached, they press a designated pause button. The actual position is then recorded to calculate the AE. This method exclusively relies on advanced isokinetic systems (49). From a psychometric perspective, relevant literature indicates that active repositioning tests generally yield superior test–retest stability and significantly lower absolute errors compared to passive repositioning modalities (40, 50). A primary advantage of the passive repositioning test is its ability to isolate joint capsuloligamentous mechanoreceptor function by minimizing the active contractile input from muscle spindles (12, 51). Furthermore, the constant angular velocity ensures highly standardized testing conditions. However, several inherent limitations exist. The protocol relies entirely on expensive, specialized, and non-portable motorized equipment (21), which severely limits its clinical feasibility for routine screening. More importantly, passive movement cannot accurately reflect the active muscle coordination and dynamic motor control required during real-life daily activities. Consequently, given the relatively low certainty of current empirical evidence, the clinical utility of passive replication metrics for diagnostic screening or therapeutic tracking remains constrained and must be interpreted with caution.

### Motion sense

Motion sense refers to the perception of movement onset and direction (52). The motion perception threshold test quantifies the minimal detectable axial trunk rotation, typically initiating from a neutral baseline position where the subject undergoes controlled clockwise or counterclockwise rotation at a low, constant angular velocity (such as 1°/s) until the direction of displacement is actively perceived and signaled via a response switch. Hence, this modality is also referred to as the Threshold to Detect Passive Motion (TTDPM) test (53). Currently, while the TTDPM paradigm has been utilized in proprioceptive assessments for post-stroke cohorts and healthy individuals, its application within spinal testing remains limited, and it notably lacks robust evidence of reliability (54, 55). Crucially, from a psychometric standpoint, the protocol notably lacks robust evidence of longitudinal test–retest reliability within spinal populations. This methodological deficiency constrains its clinical utility for routine diagnostic screening or therapeutic tracking.

### Vibration sense

Vibration sense is assessed by determining the vibration perception threshold (VPT), the minimum intensity at which vibration is first perceived. The most used vibration instrument is a tuning fork with a frequency of 128 Hz. During the measurement, the subject is instructed to close their eyes, and the vibrating tuning fork is placed on the skin overlying the bone, allowing the vibration to propagate through the surrounding tissues. The primary sensory receptors for vibration are the Pacinian corpuscles, which are widely distributed throughout the body, making it possible to measure vibration sense at any location in the lumbar spine region (56). Regarding its psychometric properties, the discriminative validity of spinal VPT remains highly controversial within mechanical spine conditions. For instance, empirical evidence from clinical cohorts undergoing surgery for lumbar disc herniation demonstrates that postoperative vibration thresholds lack the sensitivity to differentiate between patients with and without persistent subjective sensory disturbances (57). Furthermore, while VPT is a well-established diagnostic paradigm for mapping systemic peripheral neuropathies (58). Consequently, given the low certainty of current empirical data and the lack of methodological standardization for the axial skeleton, the clinical utility of vibration testing in lumbar diagnostic screening and therapeutic tracking is severely constrained.

### Force sense

Force sense describes the perception of voluntary muscular force output, typically quantified via force reproduction paradigms utilizing high-precision load cells. Standardized protocols commonly implement target thresholds scaled to relative percentages of maximum voluntary contraction (MVC)—frequently set at 50% MVC—wherein the deviation between the target baseline and the actively replicated force output is calculated as the reproduction error (59). Despite its theoretical value, the clinical utility of force sense testing in spinal care remains profoundly restricted. Current literature on force perception is heavily skewed toward peripheral joints like the knee and elbow (60–62), leaving a substantial methodological gap regarding the axial skeleton. Consequently, due to the extreme scarcity of lumbar-specific psychometric validation and the resulting low certainty of current evidence, the clinical applicability of force replication metrics for diagnostic screening or therapeutic tracking remains constrained.

### Postural balance

Human balance is regulated by a complex sensorimotor system integrating vestibular, visual, and proprioceptive inputs (63). Consequently, the feasibility of utilizing postural balance metrics as an indirect inference—rather than a direct surrogate—of localized lumbar proprioception remains a subject of significant methodological debate. While it is widely accepted that proprioception encompasses position sense, movement sense, vibration sense, and force sense, balance itself results from the interaction of vestibular and proprioceptive sensations (64). Isolating individual proprioceptive components from this integrated output remains challenging due to limitations in objective quantification and confounding dependencies on cognitive and memory function, particularly in older adults (65). In this context, evaluating trunk postural control during unstable sitting—defined as the ability to maintain a steady trunk position on an unstable surface—has been proposed to optimize regional kinematic tracking (66). Crucially, however, even optimized trunk control metrics cannot be interpreted as specific or direct indicators of isolated lumbar proprioceptive dysfunction, as balance performance is inherently contaminated by central motor coordination, volitional trunk muscle recruitment strategies, and vestibular-visual feedback loops. Methodologically, balance perception measurement approaches are generally divided into performance-based functional assessments and laboratory-based device feedback.

#### Functional assessment

Functional performance measures include various clinical techniques developed to evaluate balance and dynamic stability. These methods include the single-leg stance (OLS) test, the Timed Up and Go (TUG) test, the Berg Balance Scale, the functional reach test, and the Tinetti Balance Test (TB). While primarily used to assess fall risk, the Tinetti Balance Test is less commonly applied in proprioception assessment (67, 68). Rather than isolating micro-sensory inputs, these modalities capture distinct macroscopic dimensions of sensorimotor performance: the OLS test quantifies static unipedal stability by recording the maximum duration an individual can maintain a single-leg posture (69); the TUG test measures dynamic mobility and transitional balance via a timed locomotor task (70); the BBS provides a cumulative index based on performance across a battery of functional positional challenges (71); and the FRT determines the margin of stability in the sagittal plane by measuring maximum anterior trunk displacement over a fixed base of support (72).

Methodological data demonstrate that symptomatic populations exhibit significant functional balance deficits compared to healthy controls, manifested as prolonged TUG completion times, severely restricted forward reach distances, and diminished unipedal stance durations (73–76). These tools are highly favored in routine clinical practice due to their exceptional feasibility, low cost, minimal equipment requirements, and strong test–retest reliability for high-throughput screening (71, 77). Crucially, however, the construct validity of functional performance tests as specific or direct indicators of localized lumbar proprioception is fundamentally constrained. Postural balance on these tasks represents an integrated, multi-sensory downstream motor output that is highly susceptible to non-proprioceptive confounding variables. Empirical data reveal that performance declines are frequently driven by paraspinal muscle extensor endurance deficits, acute nociceptive pain inhibition, attentional drift, physical fatigue, and kinesiophobia rather than pure mechanoreceptor dysfunction (69, 75, 76). Consequently, while these functional tests remain valuable, highly accessible preliminary screening instruments, their results must be framed strictly as indirect inferences of broader sensorimotor disruption, and they must be paired with direct measurement techniques to avoid the overinterpretation of balance deficits as pure proprioceptive loss.

#### Device feedback

Biomechanical feedback systems utilize specialized, high-precision instrumentation—such as three-dimensional motion capture, wearable inertial sensors, and force platforms—to objectively quantify body kinematics, postural sway dynamics, and ground reaction forces (78). By tracking multi-axial spatial trajectories and real-time fluctuations in the center of mass (COM) and center of pressure (COP), these systems generate standardized balance indexes to characterize postural control (79). From a psychometric perspective, laboratory-based device feedback provides superior measurement precision, objective quantification, and high sensitivity to subtle paraspinal sensorimotor alterations that easily escape visual clinical observation. However, from a clinical utility standpoint, the feasibility of these technological modalities is profoundly restricted. These protocols rely on expensive, non-portable, and sophisticated hardware configurations that confine their application almost exclusively to laboratory research settings, rendering them unfeasible for routine, high-throughput screening in daily clinical practice.

## Discussion

Existing literature indicates that evaluation methodologies for tracking lumbar proprioception present a diverse and highly heterogeneous landscape. These paradigms encompass a spectrum ranging from direct regional metrics—such as joint position sense (JPS) quantified via dual inclinometry, tape measurements, isokinetic dynamometers, and electronic motion capture systems—to specialized modalities like force and vibration sense, alongside downstream indirect indicators such as postural balance. To effectively translate these research findings into a practical application framework, a clear distinction must be made regarding tool selection based on specific application contexts. Based on current evidence, the inclinometer-based active joint repositioning paradigm is currently best suited for routine clinical use (20). Despite its recognized absolute measurement errors, its features of low equipment cost, excellent portability, rapid testing throughput, and minimal training requirements make it highly pragmatic and functional in busy outpatient environments (35). Although literature indicates that trunk isokinetic dynamometers are considered the most objective and accurate instruments for measuring trunk position sense (37, 80), inherent drawbacks—such as their inability to assess multi-dimensional movements and the potential confounding of tactile feedback loops—remain unavoidable (42). Conversely, three-dimensional motion capture systems are ideally suited for scientific research. Although they require complex calibration and longer data processing times, these high-fidelity devices successfully eliminate manual assessor bias and provide superior spatial–temporal resolution across multi-planar spinal kinematics.

Crucially, this context-specific division of labor between clinical and laboratory tools is fundamentally determined by the underlying psychometric non-convergence and methodological limitations inherent to direct measurement metrics. Current literature indicates weak concurrent validity between dual inclinometry and tape measurement for assessing lumbar joint position sense (81). The significant measurement errors and suboptimal test–retest reliability associated with these methods underscore the need for methodological refinement. A major confounding factor is the absence of a universally accepted “gold standard” for lumbar proprioception, which complicates the determination of absolute reliability. Furthermore, this absence of a unified benchmark is compounded by the extremely poor correlations observed between different testing modalities, Larivière et al. (82) investigated the correlations among three distinct proprioceptive modalities: movement perception threshold, active joint repositioning sense, and passive joint repositioning sense. They found no significant correlations among these measures in the lumbar spine, with only a low correlation observed even between active and passive repositioning tests. Reliability studies suggest that motion perception threshold testing demonstrates higher reliability than both active and passive repositioning tests (83). This discrepancy likely stems from repositioning tests being inherently influenced by confounding cognitive factors, such as spatial memory. However, because the motion perception threshold is typically assessed using a passive rotating seat (53), it inherently lacks active muscle engagement. Consequently, it may fail to fully capture the critical role of active paraspinal muscles, which are fundamental to dynamic spinal proprioception. The absence of active muscle contraction deprives the central nervous system of essential afferent feedback, potentially challenging its ability to detect subtle trunk position changes (84). While active muscle contraction assessments may yield superior physiological relevance compared to passive methods (85), no established active technique currently exists for quantifying motion perception thresholds. Moreover, key components such as force and vibration sense remain critically underutilized in routine lumbar assessments.

This widespread underutilization of force and vibration sense is by no means accidental; rather, it stems primarily from the fact that force and vibration testing modalities currently lack sufficient lumbar-specific validation. These methods have largely been loosely extrapolated from peripheral joint protocols (such as the knee or ankle) without adequate adaptation to the multi-segmental, overlapping innervation and deep mechanical layering unique to the spinal musculature. To bridge this methodological limitation and establish a more rigorous scientific foundation, the next phase of psychometric research should shift its focus from relative reliability metrics—such as the Intraclass Correlation Coefficient (ICC)—toward absolute reliability indicators, specifically the Standard Error of Measurement (SEM) and Minimal Detectable Change (MDC). Quantifying and analyzing SEM and MDC under standardized testing postures (such as strictly controlled sitting versus standing) remains an indispensable prerequisite for methodological refinement (86).

Using postural balance as an indirect measure offers a more holistic analysis of lumbar function, an advantage over evaluating isolated proprioceptive components. However, the indirect nature of this approach must be acknowledged, as balance results from multiple interacting factors whose influence cannot be fully isolated. Future research aimed at isolating or controlling these variables could enhance the utility of balance assessment for measuring lumbar proprioception. Overall, while this method holds promise, its application should be considered based on the specific research context. Future studies should focus on validating the reliability of this method and on developing integrated approaches that combine balance perception with other proprioception measurement techniques. Driven by recent technological breakthroughs, emerging modalities such as virtual reality (VR), functional neuroimaging (fMRI/fNIRS), and surface electromyography (sEMG) offer innovative opportunities to refine lumbar proprioception assessment (87–90).

However, regardless of whether researchers optimize traditional direct tools or adopt these advanced emerging modalities, their ultimate clinical utility hinges upon their prognostic assessment value. Determining the predictive value of these metrics for long-term clinical outcomes remains a critical frontier in this field. Existing literature demonstrates that the measurement of lumbar proprioception has already been validated for predicting fall risks or rehabilitation responses (39, 67). Furthermore, although lumbar proprioception exhibits significant cross-sectional correlations with patients’ pain severity, functional disability (such as ODI scores), recurrence rates, or surgical recovery (24, 42, 91–93), whether it can provide prospective predictions for these specific clinical outcomes warrants further rigorous longitudinal research in the future.

Several limitations of this study should be acknowledged. First, structurally, this study is a narrative review rather than a systematic review. Consequently, it lacks the rigorous algorithmic screening procedures and statistical meta-analysis data characteristic of quantitative systematic reviews, which may introduce potential subjective selection and interpretation biases. Second, the scope of this review focuses predominantly on testing methodologies and direct clinical applications, whereas the exploration of data analysis techniques and underlying foundational theoretical frameworks is relatively limited. Although various evaluation tools have been categorized, the discussions regarding advanced signal processing for data analysis and deep neurophysiological motor control mechanisms remain insufficient. Third, despite emphasizing the clinical convenience of these tools, this review does not provide a systematic or quantitative analysis of the trade-offs between measurement reliability and convenience. We only qualitatively weighed these factors and failed to construct a rigorous quantitative framework to compare psychometric precision metrics (such as ICC, SEM, or MDC) against standardized clinical feasibility indicators. Lastly, this review is restricted to articles published in English and Chinese databases, which may introduce potential publication bias and omit relevant technological advancements reported in other languages.

## Conclusion

In conclusion, while a multitude of methodologies have emerged for assessing lumbar proprioception, active joint repositioning paradigms utilizing inclinometry currently offer high clinical feasibility for routine practice. Nevertheless, given the persistent absence of a universally accepted “gold standard” and the psychometric limitations of current instruments, the objective quantification of lumbar proprioception necessitates further comprehensive exploration.
